# Supplementary material for: Comparison and calibration of MP2RAGE quantitative T1 values to multi-TI inversion recovery T1 values
Source: Magn Reson Imaging. Author manuscript; Available in PMC 2026 Apr 1. (PMC11832054; doi:10.1016/j.mri.2025.110322)
Supplement: Supplementary Material [file NIHMS2048905-supplement-Supplementary_Material.docx]

Quantitative T1 mapping is sensitive to acquisition parameters and the B1 correction factor (Supplementary Fig. 1). The lookup table for point estimate MP2RAGE demonstrates this sensitivity as well (Supplementary Fig. 2).


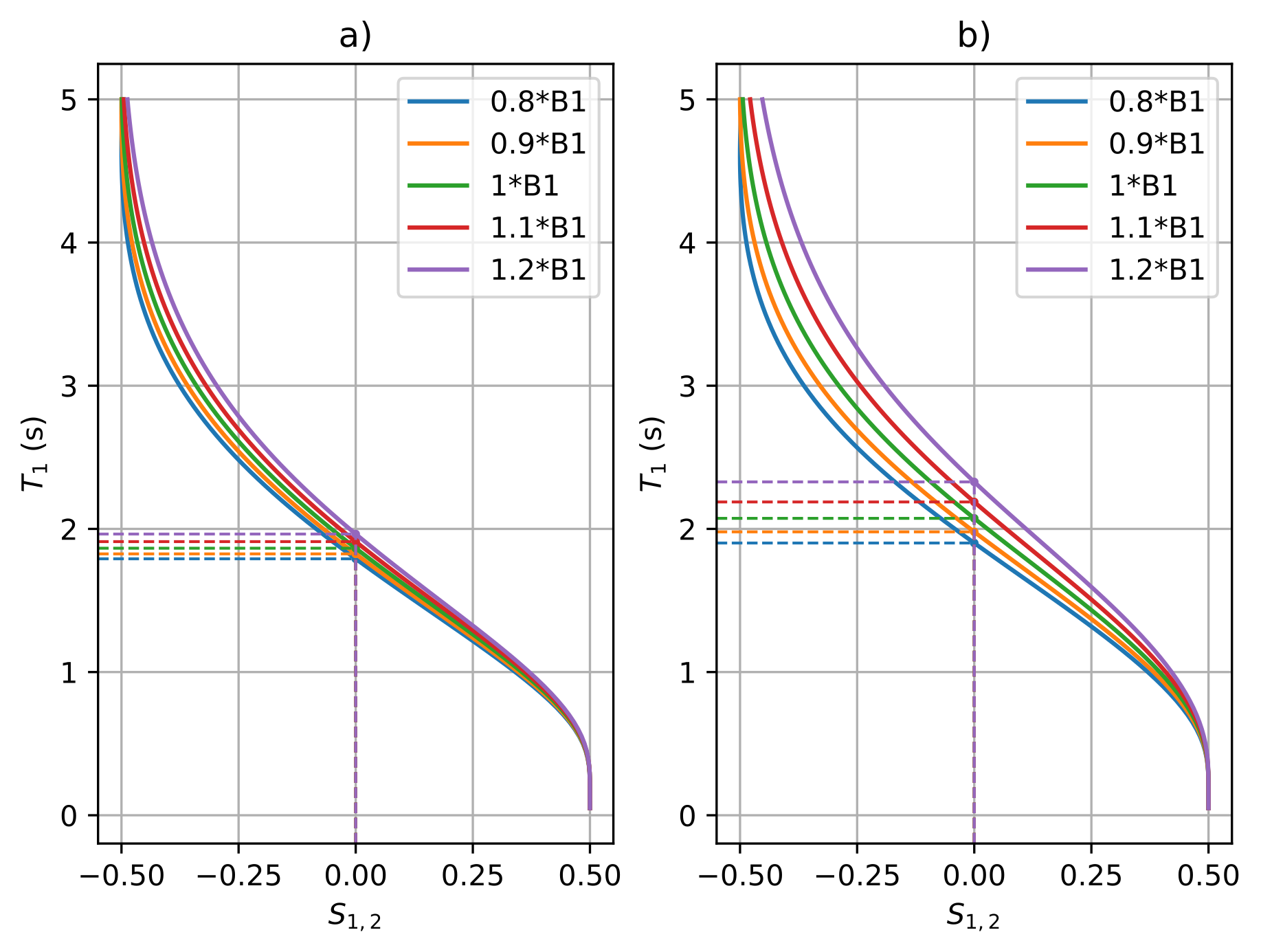


**Supplementary Fig. 1.** MP2RAGE T1 values are sensitive to the values of the acquisition parameters, as well as the B1 correction factor. Acquisition parameters are $\text{MP2RAGE}_{TR}=8.25 \text{s}$, $TR=6 \text{ms}$, $TI_{1}=1010 \text{ms}$, $TI_{2}=3310 \text{ms}$, with 225 excitation pulses, flip angles of 4 degrees, and an inversion pulse efficiency of 0.84 for a) as in this study, and $\text{MP2RAGE}_{TR}=8.5 \text{s}$, $TR=6.9 \text{ms}$, $TI_{1}=1000 \text{ms}$, $TI_{2}=3000 \text{ms}$, with 252 excitation pulses, flip angles of 5°, and an inversion pulse efficiency of 0.84 for b) as in a previous study by Choi et al. [1].

**References**


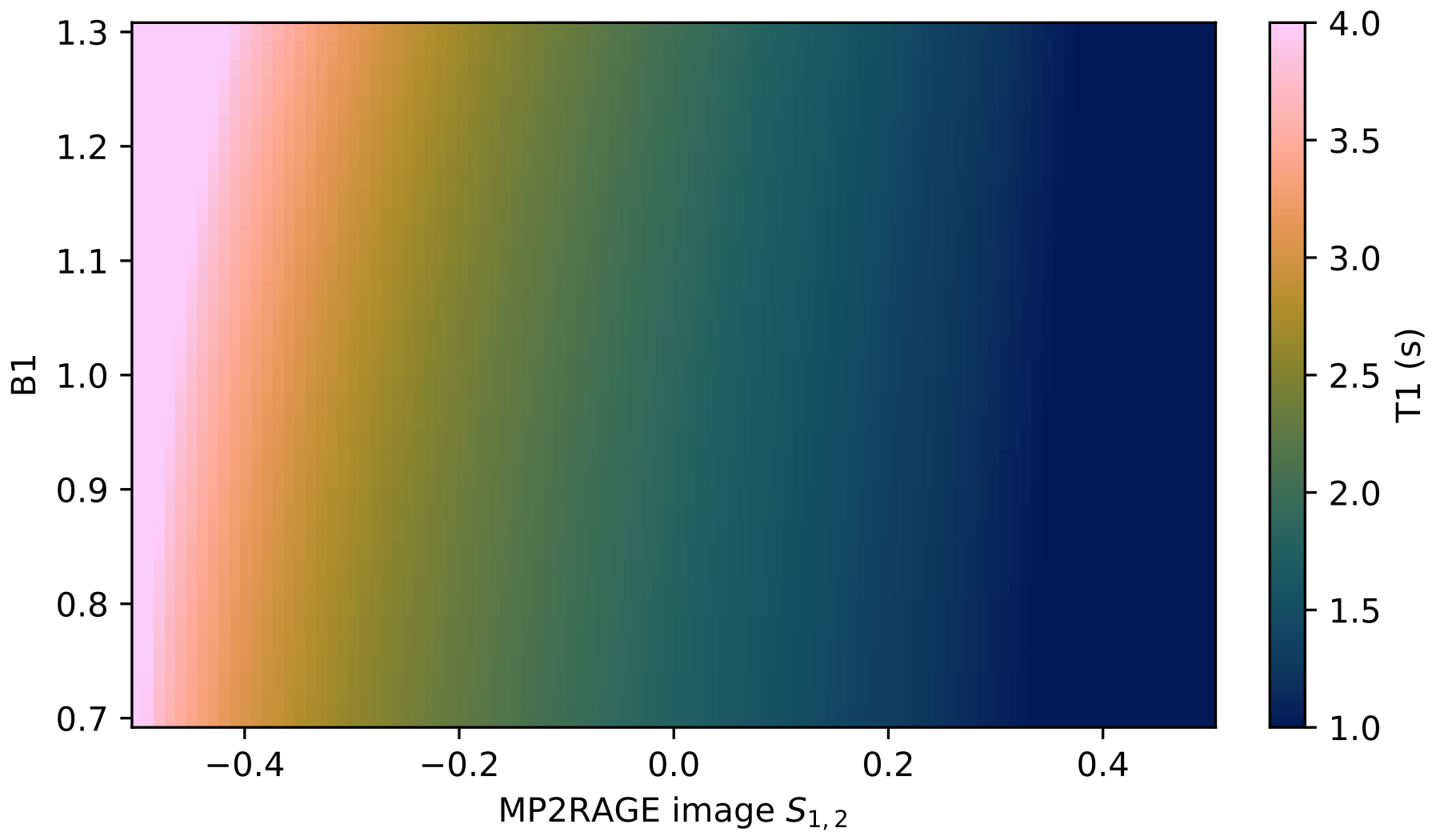


**Supplementary Fig. 2.** The lookup table for T1 from point estimate MP2RAGE T1 mapping demonstrates sensitivity to the B1 correction factor. The acquisition parameters are the same as in Supplementary Fig. 1 (a).

[1] Choi S., Spini M., Hua J., Harrison D.M. Blood-brain barrier breakdown in non-enhancing multiple sclerosis lesions detected by 7-Tesla MP2RAGE ΔT1 mapping. PLoS One 2021;16:e0249973. doi:10.1371/journal.pone.0249973.
